# Adverse childhood experiences and multimorbidity of internalising and cardiometabolic conditions in an older-age population.

**DOI:** 10.23889/ijpds.v9i5.2617

**Published:** 2024-09-18

**Authors:** Lauren Benger, LINC Consortium, Peter Holmans, Michael Owen, Mark Mon-Williams, Ioanna Katzourou, Marianne van den Bree

**Affiliations:** 1 Cardiff University; 2 University of Bristol; 3 University of Exeter; 4 University of Leeds; 5 Queen Mary; 6 University of London; 7 Wellcome Trust Sanger Institute, Cambridge

## Objectives and Approach

Alberta's largest research universities collaborated to expand COVID-19 wastewater monitoring throughout the province to regularly provide evidence of SARS-CoV-2 burden in municipalities representing 3.2 million people.

## Aims

This study investigated whether: 1) ACEs increase the risk of IC, CM and ICM-MM; 2) the effect of ACEs on risk of developing CM depends on the presence of IC and vice versa; 3) how risk differs by gender, socioeconomic status and ethnicity.

## Methods

In UK Biobank, the Childhood Trauma Screener was used to identify 157,256 participants with ACE data. Electronic healthcare records (EHR) and interview data identified those with anxiety or depression (IC) and those with hypertension, obesity, type 2 diabetes, dyslipidaemia or chronic kidney disease (CM). Multivariate logistic regression models were employed to find associations between ACEs and IC, CM and ICM-MM. Covariates included age, gender, ethnicity, socioeconomic status, diet, alcohol intake, smoking behaviour and physical activity.

## Results

All ACEs significantly increase risk for IC (all ACEs, p = <2e-16). Emotional neglect, physical neglect and emotional abuse increase risk of CM (e.g. physical neglect, OR = 1.43, CF = 1.30-1.58, p = 1.88e-13). ACEs appear to increase IC and CM risk independently.

## Conclusions

The effects of ACEs last well into adulthood and confer risk for IC and, sometimes, CM. ACE history should be included in routine primary care and the development of therapeutic interventions tailored to older adults with ACEs is key to reducing poor health.

